# A Critical Review on Health Promoting Benefits of Edible Mushrooms through Gut Microbiota

**DOI:** 10.3390/ijms18091934

**Published:** 2017-09-08

**Authors:** Muthukumaran Jayachandran, Jianbo Xiao, Baojun Xu

**Affiliations:** 1 Programme of Food Science and Technology, Division of Science and Technology, Beijing Normal University–Hong Kong Baptist University United International College, No. 28 Jinfeng Road, Tangjiawan, Zhuhai 519085, Guangdong, China; jmkbio@uic.edu.hk; 2 State Key Laboratory of Quality Research in Chinese Medicine, Institute of Chinese Medical Sciences, University of Macau, Macau, China

**Keywords:** mushroom, prebiotics, gut microbiota, anti-diabetic, anti-cancer

## Abstract

Mushrooms have long been used for medicinal and food purposes for over a thousand years, but a complete elucidation of the health-promoting properties of mushrooms through regulating gut microbiota has not yet been fully exploited. Mushrooms comprise a vast, and yet largely untapped, source of powerful new pharmaceutical substances. Mushrooms have been used in health care for treating simple and common diseases, like skin diseases and pandemic diseases like AIDS. This review is aimed at accumulating the health-promoting benefits of edible mushrooms through gut microbiota. Mushrooms are proven to possess anti-allergic, anti-cholesterol, anti-tumor, and anti-cancer properties. Mushrooms are rich in carbohydrates, like chitin, hemicellulose, β and α-glucans, mannans, xylans, and galactans, which make them the right choice for prebiotics. Mushrooms act as a prebiotics to stimulate the growth of gut microbiota, conferring health benefits to the host. In the present review, we have summarized the beneficial activities of various mushrooms on gut microbiota via the inhibition of exogenous pathogens and, thus, improving the host health.

## 1. Introduction

For countless ailments mushrooms have been used for several thousands of years. Initially, mushrooms were known to be only a source of food but, later, their medicinal properties were discovered [1]. The use of mushrooms dates back to the ancient Egyptians and ancient Chinese cultures to promote general health and longevity. The early record of the Materia Medica shows evidence of using mushrooms for treating diseases. The number of mushrooms identified to date represents only 10% of total mushrooms assumed to exist [2]. Mushrooms rich in polysaccharides, especially β glucans, can stimulate the immune system and provide the beneficial properties to medicinal mushrooms when compared to the other mushrooms. Mushrooms have high protein content (up to 44.93%), vitamins, fibers, minerals, trace elements, and low calories and they lack cholesterol [3]. Mushrooms offer significant vital health benefits, including antioxidants, cholesterol-lowering properties, anti-hypertensive, anti-inflammatory, liver protection, as well as anti-diabetic, anti-viral, and anti-microbial properties.

The prebiotics depress endogenous pathogens found within the gastrointestinal (GI) tract, allowing increased competency of the immune system to resist exogenous pathogens [4]. Prebiotics are food ingredients (such as mushroom) that can stimulate the growth of beneficial microbiota. Oligosaccharides and fibers are the major constituents of prebiotics. The recent trend in food science and technology has shown the association of prebiotics to modulate the human gut microbiota and attenuate several disease conditions such as diabetes, obesity, and cancer. The important sources of prebiotics in mushrooms are non-digestible mushroom polysaccharides which can inhibit pathogen proliferation by enhancing the growth of probiotic bacteria in the gut [5]. The gut microbiota can contribute to the onset of several metabolic dysregulations, leading to inflammation in the intestine, liver, and brain. Microbiota also regulates the energy metabolism [6].

## 2. Composition of Mushrooms

Mushrooms contain bioactive polysaccharides and essential amino acids, as well as minerals, such as such as calcium, potassium, magnesium, iron, and zinc. An interesting study revealed that the protein content in dry mushrooms was 228 and 249 g/kg dry matter (DM) [7]. Another important constituent of the mushrooms are carbohydrates, which constitute about one-half of mushroom DM. Carbohydrates play a significant role in the medicinal properties of mushrooms through their immune-stimulating β glucans, along with other polysaccharides [8]. Compared with the protein and carbohydrate contents, contents of total lipids (crude fat) are low, ranging mostly from 20 to 30 g/kg DM. Mushrooms contain various elements, in particular, potassium as the prevailing element. The compositions of many trace elements vary widely among species. The normal content of ascorbic acid is 150–300 mg/kg DM. B-group vitamin contents of thiamine (1.7–6.3 mg/kg), riboflavin (2.6–9.0 mg/kg), pyridoxine (1.4–5.6 mg/kg), and niacin (63.8–83.7 mg/kg) were determined in four dried common cultivated species. The average ergosterol content was 1.98 mg/g, the average vitamin D_2_ content was 16.88 µg/g, and vitamin B2 content was 12.68 µg/g in 35 different mushrooms. In addition, vitamin D_2_ content was increased in mushrooms followed by ultraviolet-C (UV-C) radiation [9]. There exists a consensus that phenolics—in particular, phenolic acids—are the major active component in mushroom. Phenolic acids can be divided into two major groups: hydroxy derivatives of benzoic acid and *trans*-cinnamic acid. Within the former group, protocatechuic, gentisic, *p*-hydroxybenzoic, gallic, vanillic, and syringic acids have usually been detected in mushrooms [10]. An interesting study on edible mushrooms commonly consumed in China revealed that mushrooms possess substantial antioxidant activity and the strongest metal chelating ability by virtue of their phenolic composition, in particular, gallic acid [11].

## 3. Medicinal Properties of Mushroom

Mushrooms are significant as a medicinal food. In fact, many mushrooms have long been used throughout Asia for medicinal purposes. Mushrooms possess antioxidant activity, anti-hypertensive activity, hypocholesterolemic activity, liver protection, as well as anti-inflammatory activity, anti-diabetic activity, anti-viral activity, and anti-microbial activity [12]. We are going to discuss some of the common mushrooms with medicinal properties and proven to be beneficial for improving health status (Table 1).

### 3.1. Ganoderma

Reishi is an edible medicinal mushroom that has been used for various healing abilities for several decades; it possesses a strong anti-inflammatory function and is tied to longevity, better immune function, and mental clarity [13]. It is generally referred as the king of mushrooms. The common genus of this mushroom is *Ganoderma* and closely-related species include *Ganoderma lucidum*, *G. tsugae*, and *G. lingzhi*. The total triterpenes in *G. lucidum* induce apoptosis in MCF-7 cells and attenuate Dimethylbenz[a]anthracene (DMBA) induced mammary and skin carcinomas in experimental animals [14]. *G. lucidum* polysaccharides also protect fibroblasts against UVB-induced photoaging [15]. *G. lucidum* also showed strong anti-inflammatory activity and it acted as an immunomodulator in inflammation induced by a high-cholesterol diet [16]. The recent advancement in research has shown the link between gut microbiota and treatment of various ailments. The constituents of the *G. lucidum* make it one of the important prebiotics used to increase the bacterial flora. In particular it is rich in polysaccharides, terpenoids, and total phenols. The prebiotic action of *G. lucidum* should be due to the presence of several polysaccharides; a recent study has isolated the high and intermediate polysaccharides and shown to be responsible for its prebiotic action. The specific type of polysaccharide present is the β-d-glucan polysaccharide. There are several types of polysaccharides present in *Ganoderma lucidium* polysaccharides A, B, and C in a ratio of 2.5:72.5:25. The chief sugars found in polysaccharides are rhamnose, d-galactose, and glucose and galactose. *Ganoderma* also contains water-soluble polysaccharides with anti-tumor properties. Polysaccharides BN3C, 4 (glucose and arabinose at molar ratio of 4:1) found in *Ganoderma* can boost and synthesize the metabolism of nucleic acid protein. The polysaccharides, G-I (β-d-glucan) and GL-1, for example, have both been shown to inhibit sarcoma. A fermentation study has found that fermentation of *Ganoderma lucidum* extracts have shown prebiotic ability of polysaccharides in increasing the number of *Bifidobacteria*. The potential prebiotic effect of the *G. lucidum* extract in batch-culture fermentation based on increments in the growth of bacteria used (0.4–1.5 log10 CFU/mL) after 18 h of fermentation [17]. Supplementation of *G. lucidum* polysaccharide strain S_3_ (GLPS_3_) increased the relative abundance of the beneficial bacteria such as *lactobacillus*, *roseburia*, and *lachnospiraceae* [18]. GLPS_3_ inhibits pancreatitis through microbiota regulation. In a similar way, two other species of reishi mushroom possess several pharmacological properties, including antioxidant [19], antitumor [20], and hepatoprotective activities [21]. In an interesting study ethanolic extract of *G. lucidum* showed an appreciable amount of antioxidant compounds and also good free radical scavenging effects of different free radicals. The study shows that *G. lucidum* compounds can be better antioxidant supplements for nutrients [22]. This may be due to the rich phenolic contents of *G. lucidum*.

### 3.2. Chaga Mushroom

Chaga mushroom is another promising candidate in the field of medicinal mushrooms. The major constituents are betulinic acid derivatives and melano-glucan complexes, and chaga have traditionally been boiled to make a tea, which is drunk to treat a range of conditions, including cancers, viral and bacterial infections, and gastro-intestinal disorders [23,24]. *Inonotus obliquus* belongs to higher basidiomycetes of chaga medicinal mushrooms. *Inonotus obliquus* presented protective effects against the oxidative stress in liver induced by *tert*-butyl hydroperoxide in primary-cultured rat hepatocytes. The above said property maybe due to its ability to scavenge the free radicals and thereby it inhibits the leakage of liver marker enzymes as a result of liver damage [25]. The high total phenolic contents maybe the reason for its strong antioxidant activity. Like several other mushrooms, *I. obliquus* also possesses anti-cancer activity. The ergosterol peroxide from *I. obliquus* exhibits anti-cancer activity by down-regulation of the β-catenin pathway in colorectal cancer and it shows that it down-regulated β-catenin signaling, which exerted anti-proliferative and pro-apoptotic activities in colorectal cancer (CRC) cells. This proves that *I. obliquus* can be developed as promising medicine to treat colon cancer [26]. With context to this; another study shows that ethanolic extract of *Innotus obliquus* induces G1 cell cycle arrest in HT-29 human colon cancer cells [27]. The biological activity of the *Inonotus obliquus* is mainly due to the presence of several polysaccharides, the polysaccharides of *Inonotus obliquus* mainly constitutes the following sugars: rhamnose, arabinose, xylose, mannose, glucose, and galactose. An interesting study finds that *Inonotus obliquus* polysaccharide (IOP) contains polysaccharide content of 98.6%, and Man, Rha, Glu, Gal, Xyl, and Ara in a ratio of 9.8:13.6:29.1:20.5:21.6:5.4 as monosaccharide. The study reveals that IOP induce changes in the gut microbiota and increased the Bacteroidetes at the phylum level, and brings the changes towards a healthy bacterial profile. The experiment was carried with three different doses of IOP 0.1, 0.2, and 0.4 g/kg/day. The result of the study states that the predominant phylum was Bacteroidetes, normal control (NC) shows 65.05%, 47.47% in model control (MC) group. The composition of Bacteroidetes was increased about 4.55%, 9.56%, 17.48%, and 20.81% in IOP-L, IOP-M, IOP-H, and Qingyilidan granule treated (PC) groups, respectively. The PC group (Qingyilidan granule) is the standard compared with the three different doses of IOP [28].

### 3.3. Coriolus versicolor

Like other mushrooms, *Coriolus* is also rich in polysaccharides and used as traditional medicine to treat cancer, AIDS, and some fungal infections. *C. versicolor* shows in vivo and in vitro anti-tumor and anti-metastasis effects on mouse mammary 4T1 carcinoma [29]. A remarkable immunomodulatory effect was reflected by the augmentation of IL-2, 6, 12, Tumor necrosis factor-α (TNF-α), and interferon-gamma (IFN-γ) productions from the spleen lymphocytes of *C. versicolor*-treated tumor-bearing mice. Ternatin, a cyclic peptide isolated from corioulus versicolor, and its derivative found to suppress hyperglycemia and hepatic fatty acid synthesis in diabetic Kuo Kondo yellow obese (KK-A(y)) mice. The main finding of this study proves that *C. versicolor* has lowered glucose, and triglycerides significantly (*p* < 0.05) and Sterol regulatory element-binding protein-1c (SREBP-1c) mRNA level in hepatoma 1-6 (Hepa1-6) hepatocyte cells was reduced, but not significantly [30]. The SREBP-1c is an insulin-dependent molecule that regulates de novo lipogenesis.The positive regulation on the SREBP-1c can directly regulates hyperglycemia and fatty acid synthesis via insulin signaling. Polysaccharopeptide (PSP) is the polysaccharide present in *Coriolus versicolor*. It is a heteropolysaccharide with β-1,3-glucan with β-1,6 branches. The previous study has also reported that PSP modified *Bifidobacterium* spp. and *Lactobacillus* spp. and by that it regulates the human microbiome [31]. Another study also reported that polysaccharopeptide (PSP) from *Trametes versicolor* can alter the bacterial flora. In this experiment they have healthy human for a clinical trial. In PSP group provided 1200 mg, three times daily on an empty stomach during days 1 to 14. The results of the study states that PSP regulated microbiome composition by eliciting host responses that, in turn, regulated the microbiome [32]. A recent study showed the chemical analyses of *Coriolus versicolor* extract revealed a high amount of total phenolics in *C. versicolor*, 25.8 ± 1.4 mg·g^−1^ [33]. It also shows a strong antioxidant activity in the experiment.

### 3.4. Maitake

Maitake with polysaccharides as principle constituents has been reported to strongly interact with the immune system. The mushroom *Grifola frondosa* contains starch, natural oligofructoses, fructo-oligosacharides (FOS), lactulose, galactomannan, and indigestible polydextrose, indigestible dextrin and β-glucan. *Grifola frondosa* is a species of maitake and its d-fraction is rich in proteoglucan, which has long been exclusively attributed to their immune-stimulatory capacity. In particular, it decreases cell viability, increases cell adhesion, and reduces the migration and invasion of mammary tumor cells, generating a less aggressive cell behavior in a murine model of breast cancer [34]. *G. frondosa* also inhibits hepatocellular carcinoma by inhibiting proliferation, inducing cell cycle arrest, and inducing apoptosis in Hep3B hepatoma cells [35]. *G. frondosa* also shows anti-cancer activity on B16 melanoma cells [36]. *Cordyceps* is an important mushroom of this group can benefit via metabolic efficiency increase (fat metabolism) and helps to prevent the host from viral infections by its ability to synthesize nucleoside derivatives.

## 4. Gut Microbiota-Associated Health Benefits

Human gut microbiota contains more than ten trillion microorganisms, with 1000 species of known bacteria, with more than 3 million genes (150 times more than human genes) [37]. The human gut microbiota has become the subject of extensive research in recent years and our knowledge of the inhabitant species and its functioning increased [38]. The normal human gut microbiota comprises two major phyla, namely Bacteroidetes and Firmicutes. The microbiota of the gut helps to digest the foods which cannot be digested by stomach and intestine enzymes. It plays an important role in the immune system, performing a barrier effect. Gut barrier function is defined as the ability of the gut to protect the gut from harmful substances and control the intake across the mucosa. The barrier function is classified into physical (mucous layer, intestinal epithelial cells), chemical (gastric acid, digestive enzyme, and bile acid), biological (lymphocytes and immunoglobulin A), and immunological barriers (intestinal flora). It helps with the production of some vitamins (B and K). A regular healthy balanced diet has been shown to maintain a stable and healthy gut microbiota and reduce the risk of numerous diseases [39]. The gut microbiota largely derives their nutrients from dietary carbohydrates. The link between diet, gut microbiota, and health has been elegantly shown in animal models [40]. Animal diets changed from low fat/fiber rich plant diets to high fat/high sugar diets showed a significant decrease in Bacteroidetes phylum with an increase in Bacilli and Erysipelotrichi from the Firmicutes phylum [41].

There are many recent studies focusing on health benefits of gut microbiota; for example, a study reveals that prebiotics can regulate the gut microbiota plays a significant role in regulating non-alcoholic fatty liver disease (NAFLD) [42]. A recent study shows that the gut microbiota plays a protective role in the host defense against pneumococcal pneumonia [43]. Generally, the microbiota can be activated in favor of host health by various factors, such as probiotics (indicating microorganisms stimulate microbiota), prebiotics (food compounds rich in oligosaccharides or polysaccharides), and synbiotics (a combination of probiotics and prebiotics). Prebiotics research is of vital significance in studying the health benefits of gut microbiota [44].

Gut microbiota performs several functions which proven to be beneficial to the host including following aspects.

Metabolism of various nutrients: members of the genus *Bacteroides*, known to be a cardinal organism that interferes with carbohydrate metabolism—perform this by expressing enzymes such as glycoside hydrolases, glycosyl transferases, and polysaccharide lyases. The gut microbiota has also been shown to impart a positive impact on lipid metabolism by suppressing the inhibition of lipoprotein lipase activity in adipocytes [45]. The gut microbiota is rich in protein-metabolizing enzymes that function via the microbial proteinases and peptidases in tandem with human proteinases. Another major metabolic function of the gut microbiota is the synthesis of vitamin K and several components of vitamin B [46].

Drug metabolism: the importance of the gut microbiome in determining not only overall health, but also in the metabolism of drugs and xenobiotics, is rapidly emerging. Intestinal microbiota-mediated drug and toxicant metabolism is an unexplored [47], but vital, field of study in pharmacology and toxicology.

Regulation of immune system: the gut microbiota contributes to gut immunomodulation in tandem with both the innate and adaptive immune systems [48]. During the prolonged co-evolution, bacteria and its host developed some interactions governed by the host immune system. In response to intestinal bacteria or their metabolites, a variety of innate immune cells promotes or suppresses T cell differentiation and activation [49]. Some commensal bacteria or bacterial metabolites enhance or repress host immunity by inducing regulatory T cells. The intestinal epithelial cells between host immune cells and intestinal microbiota contribute to the separation of these populations and modulate host immune responses to intestinal microbiota [50].

## 5. Role of Mushrooms as Prebiotics in Improving the Host’s Health

Prebiotics are substances that induce the growth or action of microorganisms (e.g., bacteria and fungi) that contribute to the well-being of their host [51]. Prebiotics are identified based on the composition of fibers in them. Some of the commonly-known prebiotic foods are as follows: raw chicory root (64.6%), raw Jerusalem artichoke (31.5%), raw dandelion greens (24.3%), raw garlic (17.5%), and raw onion (8.6%). Apart from those mentioned above, mushrooms are also considered a potential source of prebiotics as they contain different polysaccharides, such as chitin, hemicellulose, mannans, α- and β-glucans, galactans, and xylans [52]. Mushrooms were found to play a vital role in immunoregulating pneumococcal pneumonia, atherosclerosis, and antitumor activities. In a recent study, researchers have found that white button mushrooms (WB mushrooms) increase microbial diversity and accelerate the resolution of *Citrobacter rodentium* infection in mice [53]. Specifically, WB mushrooms were reported to stimulate a local inflammatory response, the production of catecholamines, and their metabolites, and changed the composition of the gut flora. The results of their study provide information on biological changes that occur upon WB ingestion are likely to include direct stimulation of the innate immune systems that produce inflammation and affect the composition of the gut flora which improves GI health by limiting the damage that occurs following injury or infection. Another interesting study provides evidence for hypocholesterolemia properties and prebiotic effects of Mexican *Ganoderma lucidum* in C57BL/6 mice. In brief, the study explains significant reduction in lipogenic gene expression (*Hmgcr*, *Fasn*, *Srebp1c*, and *Acaca*) and genes responsible for reverse cholesterol transport (*Abcg5* and *Abcg8*), as well as an increase in *Ldlr* gene expression in the liver and delineate a new source of bioactive compounds with hypocholesterolemic and prebiotic effects [54].

*Ganoderma lucidium* (GL) is a frequently mentioned mushroom that has been reported to reduce obesity in mice by modulating the composition of gut microbiota. GL reduces body weight, inflammation, and insulin resistance in mice fed a high-fat diet. The GL not only reverses gut dysbiosis—as indicated by the reduced Firmicutes/Bacteroidetes ratios and endotoxin-bearing Proteobacteria levels—but also alters the intestinal barrier probity and attenuates endotoxemia. The results confirm that GL can be used as a prebiotic agent to prevent gut dysbiosis and obesity-related metabolic disorders in obese individuals. Mushrooms are shown to improve the antioxidant status via microbiome alterations. The consumption of *Agaricus bisporus* mushroom affects the intestinal microbiota composition, performance, and morphology, and antioxidant levels of turkey poults. The results of this study state that *A. bisporus* is able to improve both growth performance and antioxidant activity of turkey poults and it also significantly increased the numbers of lactic acid-producing bacteria and improved the condition of the intestine [55].

Gut microbiota composition has been reported to alter the gut barrier, affect adipose tissue proliferation, and affect energy metabolism, all of which can be changed through the use of prebiotics [56]. *Lentinula edodes*-derived polysaccharide alters the spatial structure of gut microbiota in mice; in brief L2 treatment decreased the gut microbiota’s diversity and evenness in the intestine, particularly in the colon and cecum [57]. Other populations also changed in response to L2 treatment include Proteobacteria, Acidifaciens, *Bacteroides*, *Helicobacter suncus*, and *Alistipes*. Recently some researchers have evaluated the prebiotic properties of edible mushrooms, the selected mushrooms; *Pleurotus ostreatus*, *P. sajor-caju*, and *P. abalonus* represent the bifidogenic effect which can stimulate the growths of *Bifidobacterium bifidum* TISTR 2129, *B. breve* TISTR 2130, *B. animalis* TISTR 2195, and *B. longum* TISTR 2194 [58]. This study acknowledges that these mushrooms should be studied in future for their assistance in improving hosts health via its prebiotic properties. It is evident that in the above studies that mushroom can act as potential prebiotics to improve the microbiome in favor of host health. There are about 380 or more species of mushrooms that are proven to possess medicinal properties, so a large number of prebiotic sources may be encountered in the future.

Mushroom polysaccharides have been suggested to be potential prebiotics. *Lentinula edodes*-derived polysaccharide rejuvenates mice in terms of immune responses and gut microbiota. L2 reverses the gut microbiota structure, such as the reduced ratio Firmicutes/Bacteroidetes, the increased Bacteroidia, the decreased Bacilli and Betaproteobacteria, the increased Bacteroidaceae, the decreased Lactobacillaceae, and Alcaligenaceae. *Phellinus linteus* has been proved to have anti-tumor properties on skin, lung, and prostate cancer cells. *Phellinus linteus* induces changes in the composition and activity of the gastrointestinal tract microbiota that confer nutritional and health benefits to the host. The *Trametes versicolor* is a polypore mushroom. Polysaccharopeptide from *Trametes versicolor* regulates the gut microbiota to maintain the host health. *Hericium erinaceus* is a Chinese mushroom with nootropic properties that is also known as Lion’s Mane. *H. erinaceus* renders changes in the composition and activity of the gastrointestinal tract microbiota that confer nutritional and health benefits to the host.

## 6. Conclusions and Future Perspectives

The traditional medicinal system has used foods as medicines; one such kind of traditional remedy commonly used consists of mushrooms with medicinal properties. There are several edible mushrooms that have significant medicinal metabolites. These mushrooms can make better prebiotics to stimulate the gut microbiota. There are several sources of various prebiotics, such as seaweed, but mushrooms have the advantage of their easy availability and having been studied extensively, when compared to other prebiotics. Mushrooms contain various active polysaccharides and phenolic compounds make it biologically valuable. The gut microbiota comprises of trillions of bacteria that contribute to the nutrient acquisition and energy regulation [59]. The microorganisms present in the gut play an important role in the health of the digestive system, and also have an influence on the immune system. The immune tissues in the gastrointestinal tract constitute the largest and most complex fraction of the human immune system [60]. We saw earlier that gut microbiota plays an important role in various factors such as physiology, organ development, and aging. We have also discussed the beneficial effects of gut microbiota in various pathological conditions. The medicinal mushrooms can act as immunomodulatory agents to activate gut microbiota. The current review discusses the important areas in mushrooms regulated gut microbiota in the host’s health. We have discussed different mushrooms, their composition, and pharmacological properties, such as antioxidant, hypolipidemic, and atherosclerosis capabilities. We have also discussed some recent research studying the roles of mushrooms in regulating gut microbiota and the mechanism by which mushrooms regulate the gut microbiota. The exact role of the active constituents of some mushrooms are not yet documented and, in the future, a detailed research on the same would add more knowledge to the existing idea regarding the role of mushrooms in gut microbiota regulation and imparting health benefits.

As we saw earlier, microbiota play a significant role in human health and disease; often they are referred to as the “forgotten organ”. So far the research on the regulation of microbiota by various mushrooms has been insufficiently studied. Future studies on gut microbiota studies may include (1) analysis of functional composition of beneficial gut microbiota; (2) the genomic analysis of the gut microbiota and the changes happened at the genetic level of the microbiota upon the mushroom feeding (metagenomics or ecogenomics); (3) the exact mechanism by how the change in the microbial population affects the pathological conditions; (4) detailed study on the immune interaction of the host with the microbiota; (5) the role of mushrooms in balancing the microbiota to maintain the healthy host; (6) the pharmacokinetics and drug toxicity of the medicines should be studied for the effects of microbiome on it; (7) analysis of the microbial population in the early and later pathological stages; and (8) characterization of the global microbiota and how the different diets affect the microbial community. Apart from all the above directions researchers are also interested in studies related to restoring the microbial balance. An interesting study has found that donating the healthy microbiota from a donor to the host affected by *Clostridium difficile*-associated disease (CDAD) can improve the health condition. Treatment for two weeks significantly prevented the dysbiosis and the symptoms associated with CDAD were found to have disappeared [61]. Even though they achieved the beneficial effects, the host-donor compatibility and the critical risks pre- and post-transplantation of microbiota still remains unclear. This needs to be studied extensively in order to derive a proper conclusion. Overall we have summarized the updated research in the field of prebiotic-induced gut microbiota and its health benefits. The results from the above said directions may open up a great field of disease management.

## Figures and Tables

**Table 1:** List of important medicinal mushrooms and their pharmacological benefits.

Medicinal Mushroom	Active Immunomodulators	Health Benefits	Gut Microbiota Regulation
*Grifola frondosa*	MD-fraction Grifolan	The *Agaricus blazei*-based mushroom extract, andosan, protects against intestinal tumorigenesis in A/J Min/+ mice [62].	Andosan may also have influenced the composition and activity of microbiota in the A/J Min/+ mice.
*Pleurotus tuberregium*	Polysaccharides	*Pleurotus tuberregium* possesses antihyperglycemic properties and attenuated oxidative stress in diabetic rats on a high-fat diet [63].	There are possible roles of gut microbiota in the polysaccharide-induced attenuation of obesity and hyperglycemia.
*Ganoderma lucidum*	GLP(AI), Ganopoly, Ganoderans	*Ganoderma lucidum* reduces obesity in mice by modulating the composition of the gut microbiota [64].	GL has decreased Firmicutes-to-Bacteroidetes ratios. Reduced endotoxin-bearing Proteobacteria levels. It also maintains intestinal barrier integrity and reduces metabolic endotoxemia.
*Polyporus umbellatus*	Polysaccharides	Integrative fungal solutions for protecting bees [65].	Increases the intestinal microbiome to regulate host health.
*Phellinus linteus*	Polysaccharides	Anti-diabetic potential [66].	*Phellinus linteus* induces changes in the composition and activity of the gastrointestinal tract microbiota that confer nutritional and health benefits to the host.
*Trametes versicolor*	Krestin (PSK), PSP	Prevents host from diarrhea, *Clostridium difficile* infection, and inflammatory bowel disease [32].	Polysaccharopeptide from *Trametes versicolor* regulates the gut microbiota to maintain the host health.
*Hericum erinaceus*	Galactoxyloglucan–protein complex	*Hericum erinaceus* possesses anti-cancer, immuno-modulating, hypolipidemic, antioxidant and neuro-protective activities [67].	*Hericum erinaceus* renders changes in the composition and activity of the gastrointestinal tract microbiota that confer nutritional and health benefits to the host.
*Agaricus bisporus*	Polysaccharides	Anti-bacterial property [53].	White button mushrooms increase microbial diversity and accelerate the resolution of citrobacter rodentium infection in mice.
*Fomitopsis officinalis*	Polysaccharides	*Fomitopsis officinalis* acts as an insulin sensitizer in glucose tolerance tests and regulates hyperglycemia in mice with non-insulin-dependent diabetes [68].	Exact action on gut microbiota is yet to be discovered.
*Lentinula edodes*	Lentinan, KS-2	*Lentinula edodes*-derived polysaccharide rejuvenates mice in terms of immune responses and gut microbiota [69].	L2 reverses the gut microbiota structure, such as the reduced ratio Firmicutes/Bacteroidetes, the increased Bacteroidia, the decreased Bacilli and Betaproteobacteria, the increased Bacteroidaceae, the decreased Lactobacillaceae, and Alcaligenaceae.
*Fomes fomentarius*	Polysaccharides	*Fomes fomentarius* is used to cure various ailments such as dysmenorrhoea, hemorrhoids, bladder disorders, pyretic diseases, treatment of coughs, cancer, and rheumatism [70].	The exact role in regulating gut microbiota is not yet elucidated well.
*Schizophyllum commune*	Schizophyllan, Sonifilan, SPG	Used as an immune modulator [71].	The exact role in regulating gut microbiota is not yet elucidated well.

## Abbreviations

DM	Diabetes Mellitus
UV-C	Ultraviolet-C
DMBA	Dimethylbenzanthracene
MCF-7	Michigan Cancer Foundation-7 Cells
GLPS3	*G. lucidum* Polysaccharide Strain S3
IL-2	Interleukin-2
TNF-α	Tumor Necrosis Factor- α
IFN-γ	Interferon-γ
NAFLD	Non-Alcoholic Fatty Liver Disease
GI	Gastrointestinal
WB mushroom	White Button Mushroom
